# Bleb wall recession technique to repair giant bleb formation after Ahmed Glaucoma Valve implantation: a case report

**DOI:** 10.1186/s13256-019-2161-1

**Published:** 2019-07-11

**Authors:** Kaoru Manabe, Yoshifumi Ikeda, Etsuko Fujihara, Masaki Tanito

**Affiliations:** 1 Department of Ophthalmology, Shimane University Faculty of Medicine, 89-1 Enya, Izumo, Shimane 693-8501 Japan; 2 Division of Ophthalmology, Matsue Red Cross Hospital, Matsue, Japan

**Keywords:** Giant bleb, Ahmed glaucoma valve, Primary open-angle glaucoma, Anterior segment optical coherence tomography, Surgical complication

## Abstract

**Background:**

Giant bleb formation after glaucoma tube shunt surgery is a rare condition and consensus regarding its management has not been established.

**Case presentation:**

A 66-year-old Japanese man with primary open-angle glaucoma underwent implantation of an Ahmed glaucoma valve to reduce the intraocular pressure in his left eye. At 4 weeks postoperatively, he presented with a foreign body sensation in his left eye. A slit-lamp examination revealed a giant conjunctival cyst at the superotemporal quadrant and dellen formation at the corneal limbus/conjunctiva adjacent to the anterior border of the giant cyst. Ocular pain was due to a giant bleb that bulged anteriorly from the Ahmed glaucoma valve plate. Eight days after the referral, he underwent surgery to reduce the bleb volume in his left eye. To recess the bleb, the anterior edge of the dissected bleb capsule was sutured using two interrupted 10–0 absorbable sutures back to the sclera to the anterior edge of the Ahmed glaucoma valve plate. Three months postoperatively, there was no bleb around the corneal limbus, but the bleb was present around the plate.

**Conclusions:**

The surgical technique reported here can be an option to relieve dellen-associated ocular pain due to a bleb formed after tube shunt surgery.

**Electronic supplementary material:**

The online version of this article (https://doi.org/10.1186/s13256-019-2161-1) contains supplementary material, which is available to authorized users.

## Introduction

Formation of a giant bleb and the related ocular pain are rare complications after glaucoma tube shunt surgery; accordingly, no consensus regarding the management of this complication has been established. Here we report a case that was treated successfully by a bleb wall recession technique.

## Case presentation

Approval was obtained from the institutional review board of Matsue Red Cross Hospital and a written informed consent to undergo surgery and use of clinical data was provided by the patient preoperatively.

A 66-year-old Japanese man with primary open-angle glaucoma underwent implantation of an Ahmed glaucoma valve (AGV) (model FP-7, JFC Sales Plan Co., Ltd., Tokyo, Japan) to reduce the intraocular pressure (IOP) in his left eye (OS). On preoperative examination, the best-corrected visual acuity (BCVA) was 1.0 in his OS and the IOP was 22 mmHg despite instillation of a topical prostaglandin, β-blocker, and α2 agonist after failed EX-PRESS® shunt (Alcon Japan, Tokyo, Japan) placed in the superonasal quadrant. The AGV plate was placed in the superotemporal quadrant, and the tube was inserted into the anterior chamber under a partial-thickness autologous scleral flap [1, 2]. No complications developed intraoperatively. Levofloxacin 1.5% (Nipro, Osaka Japan) and betamethasone 0.1% (Sanbetason; Santen Pharmaceutical) were applied topically four times daily for 3 weeks postoperatively. At 4 weeks postoperatively, he presented with a foreign body sensation in his OS.

**Fig. 1:**
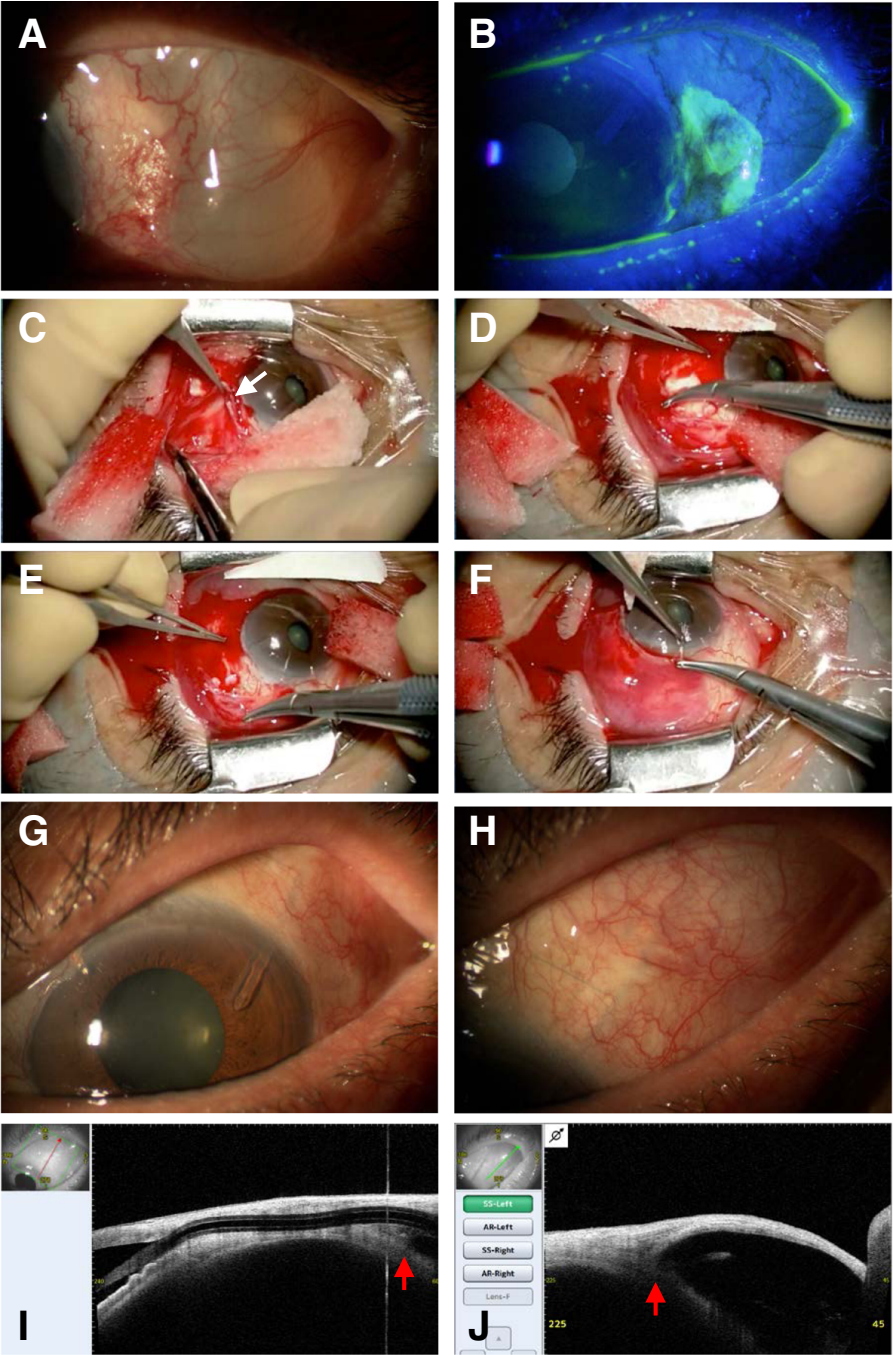
Perioperative findings. Before surgical revision, a giant conjunctival cyst (**a**) and dellen formation (**b**) stained with fluorescein are observed in the superotemporal quadrant in the left eye. During the bleb recession revision surgery, bleb capsule is dissected bluntly from the sclera and the conjunctiva (**c**, *arrow*). The anterior edge of the dissected bleb capsule is re-fixed on the sclera using two interrupted 10–0 absorbable sutures at the anterior edge of the Ahmed glaucoma valve plate (**d**, **e**). The conjunctiva is readapted with 10–0 absorbable sutures (**f**). A slit-lamp observation at 3 months postoperatively shows the findings around the corneal limbus (**g**) and the Ahmed glaucoma valve plate (**h**). Anterior segment optical coherence tomography performed 3 months postoperatively shows the findings around the Ahmed glaucoma valve tube (**i**) and the plate (**j**). The *red arrows* indicate the anterior edge of the Ahmed glaucoma valve plate


At the referral, the BCVA and IOP were, respectively, 0.9 and 20 mmHg without glaucoma medication. A slit-lamp examination revealed a giant conjunctival cyst at the superotemporal quadrant (Fig. 1a) and dellen formation at the corneal limbus/conjunctiva adjacent to the anterior border of the giant cyst (Fig. 1b). Ocular pain was due to a giant bleb that bulged anteriorly from the AGV plate. Eight days after the referral, he underwent surgery to reduce the bleb volume in his OS (Additional file 1: Video S1). Under subconjunctival anesthesia using lidocaine 2%, a limbal peritomy was performed. The bleb capsule formed by Tenon tissue was dissected bluntly from the sclera and the conjunctiva (Fig. 1c). To recess the bleb, the anterior edge of the dissected bleb capsule was sutured using two interrupted 10–0 absorbable sutures (Vicryl, Johnson & Johnson, New Brunswick, NJ) back to the sclera to the anterior edge of the AGV plate (Fig. 1d, e). The conjunctiva was readapted with 10–0 Vicryl (Fig. 1f). Postoperatively, levofloxacin 1.5% and betamethasone 0.1% were applied topically four times daily for 3 weeks. One week postoperatively, the bleb size decreased, and the ocular pain resolved. Three months postoperatively, the BCVA and IOP were, respectively, 1.0 and 14 mmHg with three anti-glaucoma medications. A slit-lamp examination showed no bleb around the corneal limbus (Fig. 1g), but the bleb was present around the plate (Fig. 1h). Anterior segment optical coherence tomography (Casia 2, Tomey Corporation, Nagoya, Japan) showed no fluid accumulation around the tube (Fig. 1i); the anterior border of the bleb was restricted at the anterior edge of the plate (Fig. 1j). At the final visit 6 months postoperatively, the BCVA and IOP were, respectively, 1.2 and 11 mmHg with three anti-glaucoma medications; a well-formed bleb was seen only around the AGV plate.

**Additional file 1:** **Video S1.** Surgical video of the bleb wall recession technique. (MP4 35099 kb)


## Discussion

The EX-PRESS® shunt and AGV were implanted in different quadrants; therefore, previous use of anti-metabolites during EX-PRESS® shunt surgery is unlikely to be associated with the giant bleb formation in this case. Previously, extension of a filtering bleb into the upper eyelid was reported in one case after Baerveldt glaucoma implant surgery [3], and in another case magnetic resonance imaging showed formation of a giant reservoir in the orbit after implantation of an AGV [4]. In previous large-scale clinical studies of tube shunt surgeries [5–7], no giant blebs were reported as a surgical complication. Thus, the current case of ocular pain due to formation of a giant bleb that caused dellen is unique in the literature. From this case, we learned that the wall of the bleb capsule can be dissected relatively easily from the conjunctiva and sclera, enabling surgical recession of the anterior border of the bleb.

## Conclusion

The surgical technique reported here can be an option to relieve dellen-associated ocular pain due to formation of a bleb after tube shunt surgery.

## Data Availability

All the relevant data are described in the manuscript.

## Abbreviations

AGV: Ahmed glaucoma valve
BCVA: Best-corrected visual acuity
IOP: Intraocular pressure
OS: Left eye
